# Sodium and Potassium Concentrations and Somatic Cell Count of Human Milk Produced in the First Six Weeks Postpartum and Their Suitability as Biomarkers of Clinical and Subclinical Mastitis

**DOI:** 10.3390/nu14224708

**Published:** 2022-11-08

**Authors:** Ryan M. Pace, Christina D. W. Pace, Bethaney D. Fehrenkamp, William J. Price, Meghan Lewis, Janet E. Williams, Mark A. McGuire, Michelle K. McGuire

**Affiliations:** 1 Margaret Ritchie School of Family and Consumer Sciences, College of Agricultural and Life Sciences, University of Idaho, Moscow, ID 83844, USA; 2 Washington, Wyoming, Alaska, Montana, Idaho (WWAMI) Medical Education Program, University of Idaho, Moscow, ID 83844, USA; 3 Statistical Programs, College of Agricultural and Life Sciences, University of Idaho, Moscow, ID 83844, USA; 4 Department of Animal, Veterinary and Food Sciences, College of Agricultural and Life Sciences, University of Idaho, Moscow, ID 83844, USA

**Keywords:** breastmilk, human milk, inflammation, lactation, mastitis, Na/K, potassium, sodium, somatic cell count, subclinical

## Abstract

The sodium (Na) concentration and the ratio of Na to potassium (K; Na/K) in human milk are used commonly as biomarkers of subclinical mastitis, but limited data exist on their relationship to and ability to predict clinical mastitis. Here, we assessed concentrations of Na, K, Na/K, and somatic cell count (SCC), a mammary health biomarker used in the dairy industry, in milk prospectively collected from both breasts of 41 women over the first 6 weeks postpartum. Although values differed over time postpartum, there were no differences in mean values between breasts. Nearly one-quarter (24%) of participants experienced clinical mastitis. Somatic cell counts >4.76 × 10^5^ cells/mL were most strongly related to development of clinical mastitis in the following week (odds ratio, 7.81; 95% CI, 2.15–28.30; *p* = 0.002), although relationships were also observed for SCC > 4.00 × 10^5^ cells/mL and Na concentration >12 mmol/L. Estimates of the prevalence of subclinical mastitis in women who never progressed to clinical mastitis differed by biomarker but ranged from 20 to 75%. Despite these findings, positive predictive values (PPV) of the biomarkers for identifying clinical mastitis were low (≤0.34), indicating additional research is needed to identify single biomarkers or composite measures that are highly specific, sensitive, and predictive of clinical mastitis in women.

## 1. Introduction

Lactational mastitis is described by the Academy of Breastfeeding Medicine as inflammation of the mammary gland that arises from a wide continuum of pathophysiological conditions [1,2]. Clinically, mastitis is defined by the presence of one or more acute signs and/or symptoms, both local and systemic, including breast engorgement not relieved by milk expression, tissue hardening, breast tenderness, diffuse or streaks of redness on the breast skin, achiness, chills, fever, and unusual nipple discharge [3]. In more severe cases, abscesses can form and may require surgical intervention, and/or antibiotics may be prescribed [1,4,5]. The etiology of mastitis is multifactorial and thought to arise due to nursing and/or hygiene practices, milk stasis, physical damage to the nipple and/or breast, and weaning. Additionally, infections by common skin commensals and/or pathobionts such as *Staphylococcus epidermidis* and *Staphylococcus aureus* have been linked to the development of mastitis [5]. Overall, clinical mastitis is estimated to occur in up to a third of lactating women and is commonly cited as a leading contributor to premature breastfeeding [6]. 

Several milk constituents have been used as biomarkers to define the presence of clinical mastitis or subclinical mastitis (generally defined as inflammation of the breast in the absence of clinical manifestations). The most common milk biomarkers include the concentration of sodium (Na) and the ratio of the concentrations of Na and potassium (K) (i.e., Na/K) [7,8,9,10,11,12]. The use of these biomarkers to discriminate between healthy and mastitis (clinical or subclinical) status can be traced back to the work of Neville et al. [13], who characterized concentrations of milk nutrients and factors associated with onset of lactogenesis and the permeability of the mammary epithelium via the opening and closing of tight junctions within the mammary epithelial junctional complex. They reported the 95% confidence intervals of the Na concentration of milk produced by healthy (nonmastitic) women to be 3–18 mmol/L. Later, work from Morton (1994) [14] defined normal milk concentrations of Na to be ≤16 mmol/L. From these and additional data, Filteau et al. [7] proposed Na concentrations >18 mmol/L and Na/K > 0.6 or >1.0 to define subclinical mastitis in women infected with HIV [7,8], reporting that 18 mmol/L Na is equivalent to Na/K > 1.0. Use of these thresholds was supported by their observations that the ratio of Na to K was correlated with concentrations of several milk-borne immune factors (e.g., secretory immunoglobulin [Ig] A, interleukin-8, lactoferrin) [7]. Since then, researchers have used these biomarker concentrations and ratios, or slightly modified concentrations (e.g., Na concentration of 12 mmol/L instead of 18 mmol/L [9]), to categorize mammary health status of lactating women into healthy, clinical mastitis, or subclinical mastitis groups to explore relationships between mammary inflammation and milk supply or the presence/concentration of nutrients or other biologically active compounds [9,10,15,16,17,18,19,20,21,22]. Surprisingly, there are little to no data comparing these biomarkers in individuals with and without clinical mastitis or assessing the accuracy and utility of these biomarkers in predicting onset of clinical mastitis. 

Representing a potentially novel biomarker of mammary health in women is the number of nucleated human (as opposed to non-nucleated, bacterial) cells present in milk; this measure is commonly referred to in the dairy industry as somatic cell count (SCC). Milk contains a variety of human cells, including mammary epithelial cells and immune cells (e.g., macrophages, leukocytes). Lower SCCs are thought to reflect a healthy mammary gland, whereas higher SCCs are thought to indicate inflammation and/or infection of the mammary gland (e.g., mastitis). Quantification of SCC in bovine milk is a commonly used method to assess mammary inflammation and milk quality in the dairy industry [23,24,25,26]. However, beyond reports of counts of individual types of cells (e.g., leukocytes) in human milk [27,28,29,30], little data exist on the use of SCC as a biomarker of mammary health in humans and its association with other mammary health biomarkers such as Na concentration and Na/K.

In the current study, we aimed to (1) characterize and compare Na and K concentrations, Na/K, and SCC in human milk in a prospective cohort of lactating women who remained healthy or spontaneously developed clinical mastitis during the first 6 weeks postpartum; (2) empirically identify thresholds for these milk constituents as biomarkers to distinguish and predict mammary health status (i.e., healthy versus clinical mastitis); and (3) estimate the prevalence of subclinical mastitis in a relatively healthy US cohort.

## 2. Materials and Methods

### 2.1. Study Design 

All study procedures were approved by the University of Idaho Institutional Review Board (#18-193, April 2019). Sample and metadata collection took place between April 2019 and December 2020 and was carried out as a longitudinal, repeated measures, prospective study. Study participants were recruited and enrolled during pregnancy or within the first week postpartum. To be eligible, participants needed to be ≥18 years of age, planning to lactate for at least 6 weeks from their date of delivery, and have no known medical conditions known to impair their ability to lactate. A total of 42 participants were enrolled, with 41 participants completing the study. One participant was lost to follow up after the first week. Participants were classified as having clinical mastitis based on the presence of one or more of the following signs/symptoms: diffuse redness/red streaks on breast, clogged duct, engorgement, breast lump or tissue hardening, breast and/or nipple pain, breast soreness/tenderness, or chills/fever accompanied by other sign(s)/symptom(s).

### 2.2. Sample Collection 

Milk samples were collected from both breasts during weekly visits over the first 6 weeks postpartum (i.e., 1, 2, 3, 4, 5, and 6 weeks ± 3 days postpartum). Participants were provided instructions and training by study personnel to perform aseptic milk collections and were asked to not feed their infant or express milk for at least 1 h prior to milk collection. Prior to milk collection, each breast was cleaned twice with prepackaged soap towelettes (PDI, Inc, Woodcliff Lake, NJ, USA) using gloved hands by participants or research personnel. Approximately 30 mL of milk was collected from each breast into sterile, single-use milk collection kits (Symphony double pump kit, Medela Inc., Baar, Switzerland) via a provided electric pump (Symphony, Medela Inc., Baar, Switzerland) or with their own electric pump using provided Symphony pump kit adaptors. Collected milk samples were placed in a cooler with cold packs, transported to the laboratory, and aliquoted for storage at −80 °C for downstream analyses. Due to regional and institutional restrictions and social distancing policies related to the coronavirus disease (COVID-19) pandemic, a small number of milk samples were immediately frozen at around −20 °C in participants’ home freezers until samples could be collected by study personnel and transported to the laboratory, thawed on ice, aliquoted, and stored at −80 °C for downstream analyses.

### 2.3. Milk Composition Analyses 

Measurements of Na and K concentrations in milk were performed using ion-selective meters. Sodium was measured with a LAQUAtwin Na-11 (model #S022, Horiba, Japan) and potassium with a LAQUAtwin K-11 (model #S030, Horiba, Japan). Ion-selective meters were used according to manufacturer recommendations as previously described [30,31]. Briefly, prior to taking measurements, ion-selective meters were conditioned and then calibrated using 150 and 2000 parts per million (ppm) Na or K standards provided by the manufacturer. Na and K measurements were made using 200–300 μL of milk. A small number of milk samples with volumes <200 μL were diluted 1:1 with Nanopure water prior to measurements and corrected for the dilution factor. Obtained values for Na and K were converted from ppm to mmol/L for downstream analysis. Quantitation of SCC in milk was performed using an automated cell counter (DeLaval cell counter, DeLaval International AB, Tumba, Sweden) according to manufacturer recommendations. Somatic cell counts were determined by drawing ~60 µL of milk into disposable cassettes which contain propidium iodide, a cell-impermeant nuclear stain, and immediately inserted into the automated cell counter for quantitation. Obtained SCCs were converted from cells/µL to cells/mL for downstream analyses. In total, 489 milk samples (244 and 245 samples from left and right breasts, respectively) had sufficient volume to measure SCC; a subset of 484 samples (242 each from left and right breasts) had sufficient volume available to measure Na and K.

### 2.4. Statistical Analyses 

All statistical analyses were performed using R (v4.0.5) [32] or Prism 9 (v9.3.1; GraphPad Software, San Diego, CA, USA). Differences in participant and pregnancy/lactation-related characteristics were tested using Wilcoxon rank sum tests for continuous data and Fisher’s exact tests for categorical data. We used lme from the R package nlme (3.1155) [33] to model differences in each milk component over time and between breasts in healthy participants; values for milk components were log_10_-transformed, week postpartum, breast, and their interaction were included as fixed effects, and participant was included as a random effect. Wilcoxon signed rank tests were used to test for differences in each milk component between breasts (i.e., healthy versus mastitic breast) within participants during clinical mastitis. Spearman correlations among biomarkers were computed using cor.test from the R stats package. The R package lme4 (v1.1-27) [34] was used to perform mixed effects logistic regression to identify optimal biomarker thresholds for classification of healthy vs. clinical mastitis status with participant included as a random effect in all models. Receiver operator characteristic (ROC) curves were generated for each biomarker using all available milk samples, and Youden’s index (*J*) was calculated (*J* = maximum [1 − sensitivity + specificity]) to identify the optimal biomarker threshold for healthy vs. clinical mastitis classification. We used glm from the R stats package and glmer from the R package lme4 [34] to perform logistic regression to analyze the dichotomous outcome of incidence of clinical mastitis within participant breasts between 2 and 5 weeks postpartum based on biomarker values above/below specific thresholds in the first week postpartum (model 1) or in the following week based on biomarker values above/below specific thresholds in the current week (model 2), respectively. In model 2, week postpartum was included as a random effect considering the potential correlations between week postpartum and participant breast. The R package ggplot2 (v3.3.6) [35] was used for data projection. Statistical significance was declared at *p* < 0.05.

## 3. Results

### 3.1. Cohort Characteristics and Incidence of Clinical Mastitis

**Figure 1:**
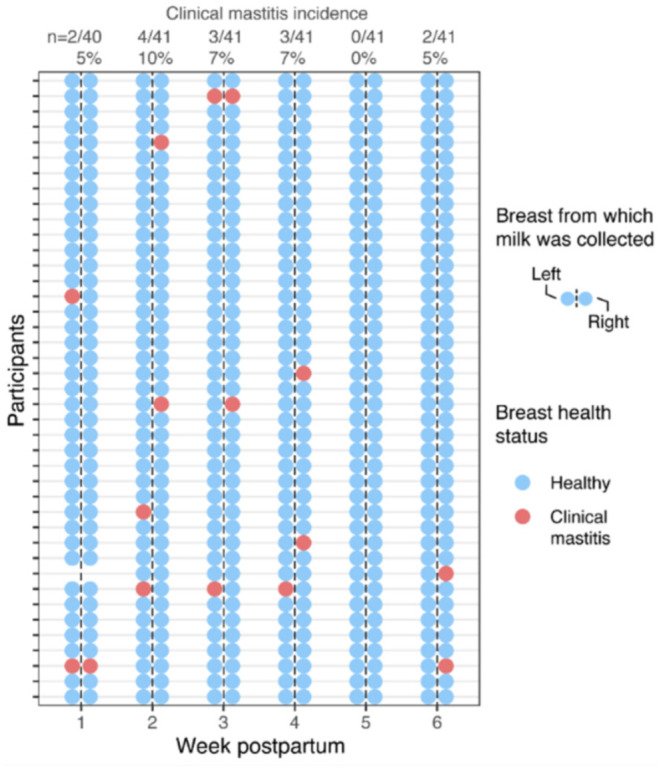
Overview of milk samples collected and incidence of clinical mastitis. A total of 41 participants were enrolled and provided weekly bilateral milk samples over the first 6 weeks postpartum. Incidence of mastitis by breast is notated by the red circles, and number and proportion of participants with clinical mastitis during each week are given at the top.

A total of 41 participants were included in the analyses. Selected participant characteristics are summarized in Table 1. Briefly, participants’ median age was 30 years (interquartile range [IQR], 28–32), with median parity of 2 (IQR, 1–3), and they delivered at a median gestational age of 40 weeks (IQR, 39–41). Additional perinatal- and lactation-related characteristics are presented in Supplementary Material Table S1. There were no detectable differences in demographic, perinatal-, or lactation-related characteristics between participants who developed clinical mastitis and those who did not, including age, history of mastitis, parity, mode of delivery, infant sex, pregnancy/delivery complications, or self-reported barriers to breastfeeding. Ten (24%) of the forty-one participants experienced clinical mastitis at least once over the course of the 6-week study, with three of these women experiencing mastitis more than once (Figure 1). These 10 participants experienced a total of 14 occurrences of mastitis, with a peak in the proportion of participants experiencing mastitis (10%) occurring in the second week postpartum. Most cases of clinical mastitis (86%, 12/14) were limited to a single breast, although two participants experienced bilateral mastitis. The occurrence of clinical mastitis was similarly distributed between the left and right breasts at 44% (7/16) and 56% (9/16) (*p* = 0.72, Fisher’s exact test), respectively. 

### 3.2. Na and K Concentrations and SCC of Milk Produced by Healthy Participants

**Figure 2:**
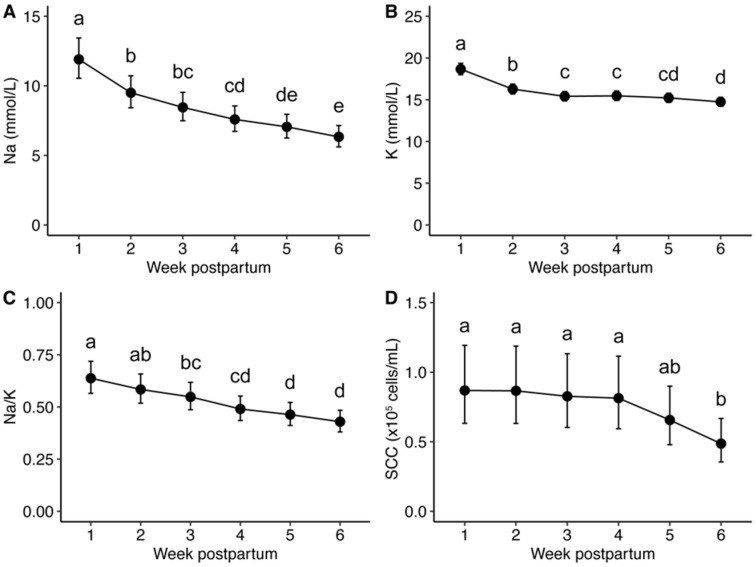
Measurements of (**A**) Na concentration, (**B**) K concentration, (**C**) Na/K ratio, and (**D**) somatic cell count (SCC) in milk produced by healthy participants during the first 6 weeks postpartum. Data are model-based estimates, means ± 95% confidence intervals. Letters denote significance groups over time, Tukey’s method *p* < 0.05.

In participants (*n* = 31) who did not experience clinical mastitis for the duration of the 6-week study period, there were no differences in Na and K concentrations, Na/K, or SCC in milk produced by left and right breasts (*p* ≥ 0.489), although differences were identified with respect to time postpartum (*p* ≤ 0.002; Figure 2, Supplementary Material Table S2). There was also no interaction between time and breast on these variables (*p* ≥ 0.444). Positive correlations between left and right breasts were observed for Na concentration (Spearman’s ρ = 0.76, *p* < 0.001), K (ρ = 0.79, *p* < 0.001), Na/K (ρ = 0.73, *P*<0.001), and SCC (ρ = 0.51, *p* < 0.001).

### 3.3. Na and K Concentrations and SCC of Milk Produced during Clinical Mastitis

**Figure 3:**
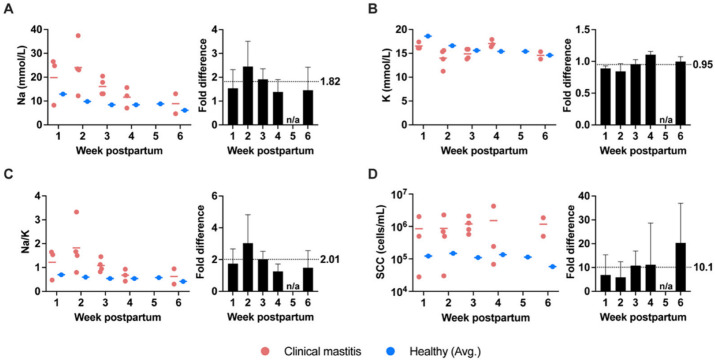
Measurements of (**A**) Na concentration, (**B**) K concentration, (**C**) Na/K, and (**D**) SCC during clinical mastitis and fold differences compared to healthy milk biomarker averages. Weekly average concentrations of each biomarker are denoted by solid horizontal bars. Fold differences were calculated by dividing the concentrations measured in mastitis samples by the average concentration of milk produced by healthy participants on a per-week level and are given as means (bars) ± standard deviations (error bars). Overall average fold difference for each biomarker is indicated by black dotted line within each respective panel; n/a, no available comparison.

Across 16 milk samples collected from the 10 participants who developed clinical mastitis during the 6-week study (Figure 1), mean values for Na and K concentrations, Na/K, and SCC in milk were 17.0 ± 8.5 mmol/L, 15.3 ± 1.7 mmol/L, 1.16 ± 0.73, and 11.06 × 10^5^ ± 11.35 × 10^5^ cells/mL, respectively (Figure 3, Supplementary Material Table S3). In comparison to values in milk collected from healthy participants, on average, Na concentration, Na/K, and SCC values during clinical mastitis were increased. Both Na concentration and Na/K were increased ~2-fold, and SCC was increased ~10-fold (Figure 3). In contrast, K concentrations were relatively similar between milk collected during clinical mastitis and milk collected from healthy participants (0.95-fold difference). Within participants who developed clinical mastitis, similar differences were observed when comparing biomarker values between paired healthy and mastitic breasts (1.8-fold difference in Na concentration; 0.98-fold difference in K concentration; 1.91-fold difference in Na/K; ~24-fold difference in SCC; Supplementary Material Figure S1).

### 3.4. Relationships among Na Concentration, K Concentration, and SCC of Milk

**Figure 4:**
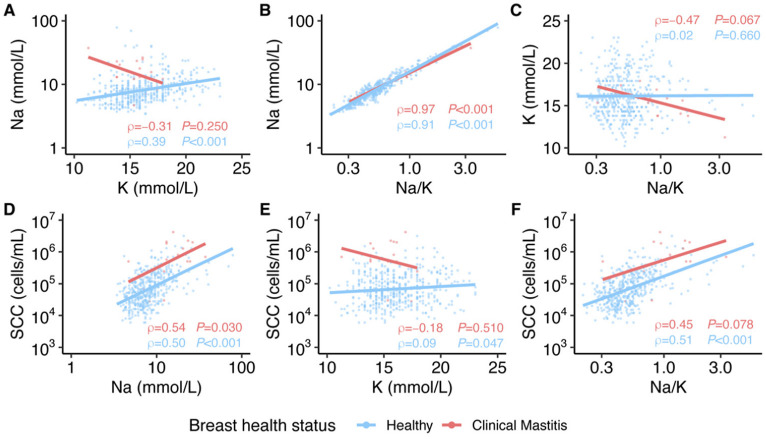
Correlations between (**A**) K and Na concentrations, (**B**) Na/K and Na concentration, (**C**) Na/K and K concentration, (**D**) Na concentration and SCC, (**E**) K concentration and SCC, and (**F**) Na/K and SCC of milk produced by healthy breasts (blue) and those with clinical mastitis (red). Lines representing linear model fits are included only for visualization.

In general, correlations among milk constituents measured and SCC in milk were positive, regardless of whether the milk was produced by healthy individuals or during clinical mastitis (Figure 4). The strongest correlations were observed between Na and Na/K (healthy: ρ = 0.91, *p* < 0.001; clinical mastitis: ρ = 0.97, *p* < 0.001; Figure 4B), followed by SCC and Na (healthy: ρ = 0.50, *p* < 0.001; clinical mastitis: ρ = 0.54, *p* = 0.030; Figure 4D), and SCC and Na/K (healthy: ρ = 0.51, *p* < 0.001; clinical mastitis: ρ = 0.45, *p* = 0.078; Figure 4F).

### 3.5. Performance and Thresholds for Classification of Clinical Mastitis Using Na and K Concentrations and SCC of Milk

**Figure 5:**
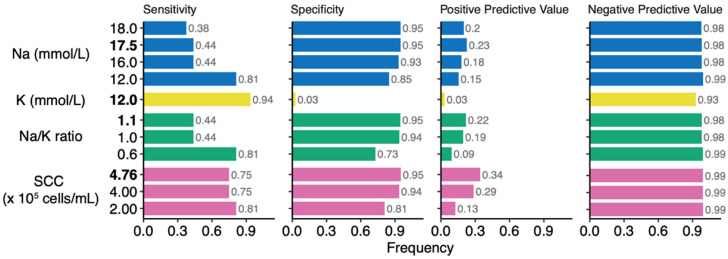
Sensitivity, specificity, positive predictive value, and negative predictive values of the different biomarkers and thresholds in classifying samples as having been produced by a breast with clinical mastitis.

Mixed effects logistic regression was used to identify thresholds for each biomarker in classifying milk samples as being collected during clinical mastitis, including >4.76 × 10^5^ cells/mL SCC, 17.5 mmol/L Na, 12 mmol/L K, and Na/K ratio of 1.1 (Supplementary Material Figure S2). The sensitivity (true positive rate), specificity (true negative rate), positive predictive value (PPV, probability that a sample classified as mastitic was indeed a mastitic sample), and negative predictive value (NPV, probability that a sample classified as non-mastitic was indeed a non-mastitic sample) for each biomarker and respective thresholds in classifying clinical mastitis were also assessed and compared to thresholds previously reported in the literature (Figure 5). Although K concentration of >12 mmol/L had the highest sensitivity (0.94) among all biomarkers and thresholds, its specificity and PPV were the lowest (0.03 for both). Somatic cell count > 2.00 × 10^5^ cells/mL, Na concentration > 12 mmol/L, and Na/K > 0.6 had the next highest sensitivities (0.81 for all) but differed with respect to the other metrics. Among biomarkers with a sensitivity of 0.81, Na > 12 mmol/L had the highest specificity (0.85); higher specificities (0.95 for all) were observed for Na concentrations > 17.5 and > 18 mmol/L, as well as Na/K > 1.1 and SCC > 4.76 × 10^5^. Somatic cell count > 4.76 × 10^5^ cells/mL had the highest PPV (0.34), followed by SCC > 4.00 × 10^5^ cells/mL (0.29). Except for the abovementioned NPV of K, the NPVs for all other biomarkers and thresholds were 0.98–0.99.

As the specificity and PPV of K concentration in classifying clinical mastitis were very low, we next focused on examining the ability of the other three biomarkers (Na concentration, Na/K, and SCC) to predict clinical mastitis. In the first model, we examined the likelihood of developing mastitis between 2 and 5 weeks postpartum based on biomarker values above/below specific thresholds in the first week postpartum (Table 2). In the second model, we examined the likelihood of developing mastitis in the following week based on biomarker values above/below specific thresholds in the current week (Table 2). Only SCC > 4.00 × 10^5^ cells/mL and >4.76 × 10^5^ cells/mL in the first week postpartum had positive relationships with future occurrence of clinical mastitis in the first 6 weeks postpartum (OR, 9.86; 95% CI, 1.05–93.60; *p* = 0.033) (Table 2). When we examined the association between Na concentration, Na/K, and SCC cut-offs and development of clinical mastitis in the following week, relationships were only identified with Na concentration and SCC (Table 2). In descending order for the likelihood of predicting mastitis in the following week was SCC > 4.76 × 10^5^ cells/mL (OR, 7.81; 95% CI, 2.15–28.30; *p* = 0.002), SCC > 4.00 × 10^5^ cells/mL (OR, 6.08; 95% CI, 1.70–21.80; *p* = 0.006), and Na concentration > 12 mmol/L (OR, 5.28; 95% CI, 1.57–17.80; *p* = 0.007).

### 3.6. Incidence and Prevalence of Subclinical Mastitis over the First 6 Weeks Postpartum

**Figure 6:**
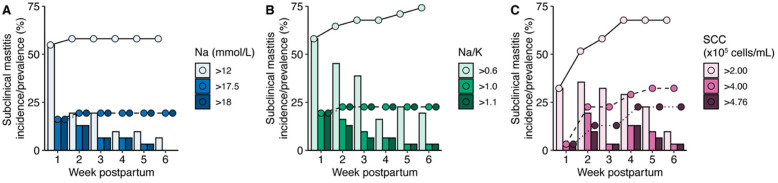
Incidence and prevalence of subclinical mastitis in healthy participants (*n* = 31) across the first 6 weeks postpartum based on (**A**) Na concentration, (**B**) Na/K, and (**C**) SCC thresholds. Subclinical mastitis was defined as exceeding biomarker thresholds that have been reported in the literature or empirically derived in this report. Bars indicate incidence of subclinical mastitis within each week, and connected points represent cumulative prevalence of subclinical mastitis over time.

Estimates of weekly incidence and cumulative prevalence of subclinical mastitis over the 6-week study were evaluated for each biomarker using model-based and literature-reported thresholds (Figure 6). Based on SCC, the incidence of subclinical mastitis in the first week ranged from 3 (SCC > 4.00 × 10^5^) to 32% (SCC > 2.00 × 10^5^); cumulative prevalence of subclinical mastitis plateaued at 67% in the fourth week based on SCC > 2.00 × 10^5^, 32% by week 5 at SCC > 4.00 × 10^5^ and 23% at SCC > 4.76 × 10^5^. Based on Na concentration > 12 mmol/L, incidence of subclinical mastitis in the first week was 52% and rose to 60% in the second week and thereafter. Based on Na/K > 0.6, incidence of subclinical mastitis in the first week was 58% and rose by approximately 3–6% each week to 74% in week 6 postpartum. There was no difference in incidence of subclinical mastitis based on the higher thresholds for Na concentration (17.5 and 18 mmol/L, 16% subclinical mastitis in the first week) and Na/K (1.0 and 1.1, 19% subclinical mastitis in the first week), both of which plateaued after the first week at a prevalence of 19% and 23%, respectively. 

## 4. Discussion

### 4.1. Na and K Concentrations, Na/K, and SCC in Milk Produced during the Early Postpartum Period

In this study we assessed weekly measurements of Na and K concentrations, Na/K, and SCC in milk collected over the first 6 weeks postpartum from women who remained healthy or developed clinical mastitis. Importantly, this study provides some of the first longitudinal data on measurements of SCC, a biomarker that has been used in the dairy industry to assess mammary health and milk quality for decades, in human milk over the first 6 weeks postpartum. We also assessed the ability of these metrics to serve as biomarkers for mammary health to predict occurrence of clinical mastitis and estimate incidence/prevalence of subclinical mastitis. 

Whereas differences in these milk constituents were identified with respect to week postpartum in women who remained healthy over the study period, there were no differences identified in these measures between breasts. However, clinical mastitis was associated with an approximately 2-fold increase in Na concentration and Na/K, and a 10-fold increase in SCC compared to healthy breasts; no difference was observed with respect to K concentration. 

Our results on the bilaterality of milk Na and K concentrations and Na/K (ρ ≥ 0.73, *p* < 0.001) are consistent with several prior reports [36,37]. Koo and Gupta (1982) found no difference in Na concentrations in milk produced by healthy breasts of 12 women between 3 and 26 days postpartum [36]. Semrau et al. (2008) examined milk Na concentrations in women with HIV during week 1, week 4, and month 4 postpartum and found Na concentrations between breasts were correlated in the first and fourth weeks, but not at month 4 [37]. Although no comparative data exist on human milk SCC during early lactation, in alignment with our Na and K concentration and Na/K results, we also found a moderately positive correlation for SCC between breasts (ρ = 0.51, *p* < 0.001). The longer-term dynamics of milk SCC remain to be determined.

### 4.2. Milk Na, K, Na/K, and SCC as Biomarkers for Clinical Mastitis

In our assessment of Na and K concentrations, Na/K, and SCC in human milk as biomarkers of clinical mastitis we identified thresholds similar to previously reported values: Na concentration of 12–18 mmol/L, Na/K of 0.6–1.0 [7,8,9,10,11,12] and SCC of 2.00 × 10^5^–4.00 × 10^5^ cells/mL [38,39,40]. Within our cohort we identified Na concentration of 17.5 mmol/L, K concentration of 12 mmol/L, Na/K of 1.1, and SCC of 4.76 × 10^5^ as optimal thresholds for clinical mastitis classification. Upon closer examination of these thresholds, we found thresholds for SCC (2.00 × 10^5^–4.76 × 10^5^ cells/mL) that consistently had some of the highest sensitivities (0.75–0.81), specificities (0.81–0.95), and NPVs (0.99). The various thresholds for Na concentration, Na/K, and SCC all had relatively high specificities (0.73–0.95) and NPVs (0.93–0.99) indicating that they had excellent abilities to identify and predict healthy breasts. Although the threshold of 12 mmol/L for K concentration had the highest sensitivity (0.94), its specificity and PPV were exceptionally low (0.03 for both). Indeed, the PPVs of all biomarkers at all examined thresholds were low. The highest PPV (0.34) was based on a SCC threshold of >4.76 × 10^5^ cells/mL, indicating 66% of the predicted clinical mastitis cases were healthy samples erroneously classified as clinical mastitis. A potential explanation for this large rate of misclassification may reflect a high incidence of subclinical mastitis (Figure 6).

We also examined the ability of Na concentration, Na/K, and SCC to predict future incidence of clinical mastitis in the first 6 weeks postpartum based on values in the first week postpartum or in the week prior to onset of clinical mastitis. We found that using thresholds of >12 mmol/L for Na concentration and SCC of >4.00 × 10^5^ in the first week postpartum were associated with increased risk of future incidence of clinical mastitis. For example, SCC of >4.00 × 10^5^ cells/mL in the first week postpartum was associated with an OR of 9.86 (95% CI 1.05–93.60). In addition, SCC thresholds of >4.00 × 10^5^ and >4.76 × 10^5^ were associated with higher incidence of clinical mastitis in the following week (OR 6.08 [95% CI 1.70–21.80] and OR 7.81 [95% CI 2.15–28.30], respectively). 

Our results on milk biomarker thresholds for clinical mastitis differ somewhat from a recent study by Furukawa at al. [41], who examined whether Na/K could serve as a diagnostic tool for clinical mastitis in a Japanese cohort of 107 participants (*n* = 55 healthy group, *n* = 52 mastitis group; samples collected <1 to 9 months postpartum). These researchers found that Na/K between 0.59 and 0.6 performed well as a diagnostic cut-off. It is likely that differences between our study population and timeline (first 6 weeks postpartum; United States, predominantly white, non-Hispanic) and those of Furukawa at al. may partially explain our different findings. Indeed, many human milk constituents vary across diverse populations [42,43,44,45,46,47,48,49,50]. However, similar to Furukawa et al. [38], we found a number of our study participants with clinical mastitis had Na concentrations (as well as Na/K and SCC) below the lowest thresholds despite having overt clinical signs/symptoms of mastitis. One explanation for this, as posited by Furukawa and colleagues, is that there may be variation in the severity of clinical mastitis [38]. An additional explanation might be that in some cases of clinical mastitis, milk secretion from inflamed mammary glands may be partially inhibited due to blockages along the ducts, resulting in biomarker measurements similar to those of milk secreted predominantly by healthy mammary glands.

The relationship between mastitis (clinical or subclinical) and milk SCC in humans is not well studied. Somatic cell count is a broad measure of nucleated cells of host origin in milk, which in humans are predominantly mammary epithelial cells [51,52]. SCC is mostly used by and reported in the dairy industry to identify subclinical mastitis at the herd level. It should be noted that the underlying somatic cell populations in bovine milk differ from human milk. The predominant cell types in bovine milk are immune cells (i.e., macrophages and lymphocytes), with elevations in SCCs during mastitis attributed to an influx of neutrophils [53,54,55,56]. Our data on differences in the values of SCC (>10-fold increase, Figure 3 and Supplementary Material Figure S1) during clinical mastitis and healthy states are similar to previous findings from our research group suggesting a 13-fold increase during clinical mastitis in a small (*n* = 14) cohort of women [57]. While we did not perform differential cell counts on the cellular subpopulations in our milk samples, it is likely that the observed increase in SCC during mastitis was also due to an increase in neutrophils. This is supported by data from Gantt et al., 2007 [29] who performed differential cell counts in milk collected from women infected with HIV and found that the quantity of neutrophils was increased in association with mastitis.

### 4.3. Estimation of the Prevalence of Subclinical Mastitis

Little data exist on the prevalence of subclinical mastitis in relatively healthy populations. Most data on estimates come from studies examining the maternal-to-child transmission of HIV or immune factors via breastfeeding [7,8,16,58]. In our study we found the cumulative prevalence of subclinical mastitis to vary based on the biomarker and threshold, ranging from 19% to 74% of women who did not develop clinical mastitis during the first 6 weeks postpartum (Figure 6). For example, the prevalence of subclinical mastitis based on a Na/K of >1.0 was 23% in our cohort, which is lower than the 43.5% in a Ghanaian cohort described by Aryeetey et al., although milk samples were collected at 3 to 4 months postpartum in the latter [18]. Our results are consistent with the findings of Rutagwera et al. [59], who reported more than 75% of women produced milk with a Na/K of ≥0.6, and more specifically 59% and 28% of women produced milk with Na/K ≥0.6 to ≤1 and Na/K ratio >1, respectively. Our results suggest that 74% of healthy women who did not develop clinical mastitis in the first 6 weeks postpartum produced milk with a Na/K >0.6 (50% and 23% of women produced milk with a Na/K >0.6 to ≤1 and Na/K ratio >1, respectively). Similarly, in these same women the incidence of subclinical mastitis based on a Na concentration >12 mmol/L at 6 weeks postpartum was 6.5% in our cohort, a slightly lower estimate than that reported by Semba et al. (15.6%) in a cross-sectional study of Malawian women at the same time postpartum [10]. Despite the lower weekly incidence of subclinical mastitis based on a Na concentration >12 mmol/L, the cumulative prevalence of subclinical mastitis in our cohort during the first 6 weeks postpartum was 58%. To our knowledge, there are no prior estimates of subclinical mastitis based on SCC. In our cohort, the estimates of subclinical mastitis based on SCC thresholds were higher than those based on the other biomarkers, with 23% and 68% of women classified as experiencing subclinical mastitis by 6 weeks postpartum based on SCC > 4.76 × 10^5^ cells/mL and >2.00 × 10^5^ cells/mL, respectively. The impact, if any, of exposure to differential levels of SCC in milk on the health of breastfed infants in relatively healthy populations is unknown, although increased SCC may be related to greater risk of HIV transmission. 

### 4.4. Relevance for Adequate Intake (AI) Values for Na and K in Early Life

For infants from 0 to 6 months of age, the most recent Institute of Medicine (now National Academy of Medicine) Adequate Intake (AI) values for Na and K are 110 mg/d and 400 mg/d, respectively [50,60]. These values were derived by multiplying the average volume of milk consumed by an exclusively breastfed infant at 6 months of age (0.78 L/day) and the average concentrations of Na and K in human milk (stated as 6.2 mmol/L and 13.1 mmol/L, respectively). Although we did not assess milk production or infant milk consumption in the current study, using the average exclusively breastfed infant milk volume consumption of 0.78 L/d suggests that infants of healthy women at each of the 6 weeks in this study are consuming approximately 213, 170, 152, 136, 127, and 113 mg/d of Na and 570, 497, 470, 473, 464, and 448 mg/d of K. Whereas the values of the latter weeks are relatively close to the AI values at 6 months, the values in the earlier weeks are well above the AI values. Assuming milk production and infant milk consumption do not change during clinical mastitis, the estimated K intake (467 mg/d) by exclusively breastfed infants during clinical mastitis would be within the range consumed by infants nursed by healthy mothers. However, assuming milk production does not decrease during clinical mastitis (as evidence suggests for subclinical mastitis [61]), we also estimate intake of Na during clinical mastitis to be nearly three times higher (304 mg/d) than the AI value. Implications, if any, for infant health are not known.

### 4.5. Limitations and Future Directions

Generalizability of the current study is limited by cohort demographics (predominantly white, non-Hispanic; United States-based), size, and relatively low number of milk samples collected during clinical mastitis. However, a key strength of the study was the prospective collection of samples from participants with potential for spontaneous development of clinical mastitis, rather than collection from participants recruited after seeking lactation support and/or treatment for mastitis. Future studies should seek to recruit sufficiently large and diverse population cohorts to further complement and extend the findings of this study. In addition, only a small subset of mammary inflammation biomarkers was examined in this study. Additional work should expand the scope of research to include other putative mammary health biomarkers (e.g., interleukin-8, transforming growth factor-β [TGF-β], lactose, lactoferrin, and lactate dehydrogenase) as well as examine composite measures of biomarkers (akin to Na/K ratios) for improving the prediction and/or use in the prevention of future mastitis.

## 5. Conclusions

Clinical mastitis was relatively common in the first 6 weeks postpartum, occurring in nearly one-quarter of study participants and associated with increased Na concentration, Na/K, and SCC in milk. Elevated SCC in milk in the first week postpartum was predictive of development of clinical mastitis within the first 6 weeks postpartum. In addition, elevated SCC or Na concentration in milk in the prior week was related to development of mastitis. However, diagnostic metrics (e.g., positive predictive value) indicate that the ability of these biomarkers to serve as prognostic biomarkers of clinical mastitis is relatively weak. Depending on the biomarker and threshold used for classification, ~20–75% of healthy participants that never experienced clinical mastitis in this study could be classified as having subclinical mastitis. 

## Figures and Tables

**Table 1:** Selected participant characteristics.

Characteristic	Total	Healthy	Mastitis	*p*
Participants, No.	41 (100)	31 (76)	10 (24)	
Age, median (IQR), y	30 (28–32)	30 (28–33)	28 (26–30)	0.082
Race, Ethnicity				0.314
American Indian/Alaskan	1 (2)	0 (0)	1 (10)	
Asian	1 (2)	1 (3)	0 (0)	
Black, Hispanic	1 (2)	1 (3)	0 (0)	
White, Hispanic	5 (12)	5 (16)	0 (0)	
White, Non-Hispanic	32 (78)	23 (74)	9 (90)	
Not reported	1 (2)	1 (3)	0 (0)	
Pre-pregnancy BMI, median (IQR), kg/m^2^	23 (21–25)	23 (21–25)	23 (22–24)	0.819
Education level				0.447
High school or less	3 (7)	3 (10)	0 (0)	
Some college	9 (22)	7 (23)	2 (20)	
Bachelor’s degree	13 (32)	8 (26)	5 (50)	
Graduate/professional degree	16 (39)	13 (42)	3 (30)	
Pets in home	25 (61)	19 (61)	5 (50)	0.714
History of mastitis	6 (15)	5 (16)	1 (10)	>0.999
Gravidity, median (IQR), No.	3 (1–4)	3 (2–4)	2 (1–4)	0.456
Parity, median (IQR), No.	2 (1–3)	2 (1–3)	2 (1–3)	0.962
Mode of delivery				>0.999
Vaginal	30 (73)	23 (68)	7 (70)	
Cesarean	11 (27)	8 (32)	3 (30)	
Gestational age at delivery, median (IQR), wk	40 (39–41)	40 (39–41)	40 (40–41)	0.463
Infant sex, female	22 (54)	19 (61)	3 (30)	0.145

IQR, interquartile range; BMI, body mass index. Values are given as numeric counts and percentages or IQR in parentheses. Percentages may not sum to 100 due to rounding. *p* values were calculated from Wilcoxon rank sum tests for continuous data and Fisher’s exact tests for categorical data, where applicable.

**Table 2:** Likelihood of developing clinical mastitis during the first 6 weeks postpartum based on Na concentration, Na/K, and SCC in milk produced during the first week postpartum (model 1) or in the week prior to mastitis onset (model 2).

	Model 1 (First Week Postpartum)		Model 2 (Previous Week)	
Biomarker	OR (95% CI)	*p*	OR (95% CI)	*p*
**Na (mmol/L)**				
>12.0	1.20 (0.28–4.92)	0.798	**5.28 (1.57–17.80)**	**0.007**
>16.0	2.19 (0.42–9.54)	0.309	2.21 (0.46–10.60)	0.322
>17.5	1.94 (0.26–9.66)	0.451	0.00 (0.00–∞)	>0.999
>18.0	1.94 (0.26–9.66)	0.451	0.00 (0.00–∞)	>0.999
**Na/K**				
>0.6	1.01 (0.23–4.11)	0.993	2.71 (0.81–9.04)	0.106
>1.0	0.85 (0.04–5.48)	0.882	1.09 (0.14–8.81)	0.933
>1.1	0.97 (0.05–6.39)	0.977	1.31 (0.16–10.60)	0.802
**SCC (×10^5^ cells/mL)**				
>2.00	1.59 (0.31–6.73)	0.543	1.97 (0.56–6.89)	0.288
>4.00	**9.86 (1.05–93.60)**	**0.033**	**6.08 (1.70–21.80)**	**0.006**
>4.76	**9.86 (1.05–93.60)**	**0.033**	**7.81 (2.15–28.30)**	**0.002**

OR, odds ratio; CI, confidence interval; significant associations are in bold.

## Data Availability

Data and materials that support the findings of this study are available upon request from the corresponding authors.

## Supplementary Materials

The following supporting information can be downloaded at: https://www.mdpi.com/article/10.3390/nu14224708/s1, Figure S1: Comparisons of Na concentrations, K concentrations, Na/K, SCC between breasts within subjects during clinical mastitis; Figure S2: Analysis of biomarkers in milk produced by women with and without clinical mastitis; Table S1: Self-reported perinatal- and lactation-related characteristics of participants who were healthy for the entire study and those who developed clinical mastitis; Table S2: Na concentrations, K concentrations, Na/K, and SCC summary measurements during the first 6 weeks postpartum in lactating women without clinical mastitis (*n* = 31); Table S3: Na concentrations, K concentrations, Na/K, and SCC summary measurements of milk samples produced during clinical mastitis in the first 6 weeks of lactation.
