# Supplementary material for: Sodium and Potassium Concentrations and Somatic Cell Count of Human Milk Produced in the First Six Weeks Postpartum and Their Suitability as Biomarkers of Clinical and Subclinical Mastitis
Source: Nutrients. 2022 Nov 8;14(22):4708. doi: 10.3390/nu14224708 (PMC9694808; doi:10.3390/nu14224708)
Supplement: Supplementary file 1 [file nutrients-14-04708-s001.zip › nutrients-2006078-supplementary.pdf]

nutrients

MDPI

Supplementary materials for

Sodium and potassium concentrations and somatic cell count of human milk produced in the first six weeks postpartum and their suitability as biomarkers of clinical and subclinical mastitis

Figure S1. Comparisons of Na concentrations, K concentrations, Na/K, SCC between breasts within subjects during clinical mastitis.

Figure S2. Analysis of biomarkers in milk produced by women with and without clinical mastitis.

Table S1. Self-reported perinatal- and lactation-related characteristics of participants who were healthy for the entire study and those who developed clinical mastitis.

Table S2. Na concentrations, K concentrations, Na/K, and SCC summary measurements during the first 6 weeks postpartum in lactating women without clinical mastitis (*n*=31).

Table S3. Na concentrations, K concentrations, Na/K, and SCC summary measurements of milk samples produced during clinical mastitis in the first 6 weeks of lactation.

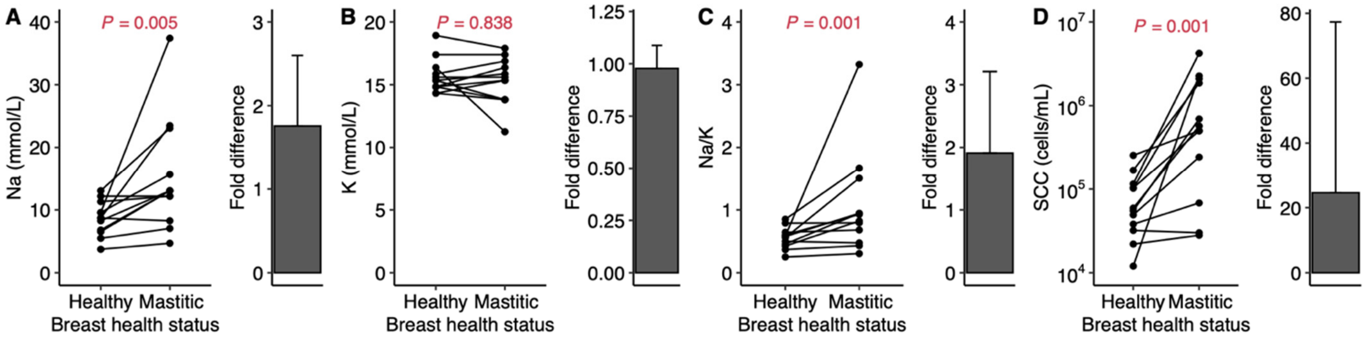

Figure S1. Comparisons of Na concentrations, K concentrations, Na/K, SCC between breasts within subjects during clinical mastitis. (A) Na, (B) K, (C) Na/K, and (D); n=12 paired healthy and mastitic breast observations. P-values were calculated from Wilcoxon signed rank tests.

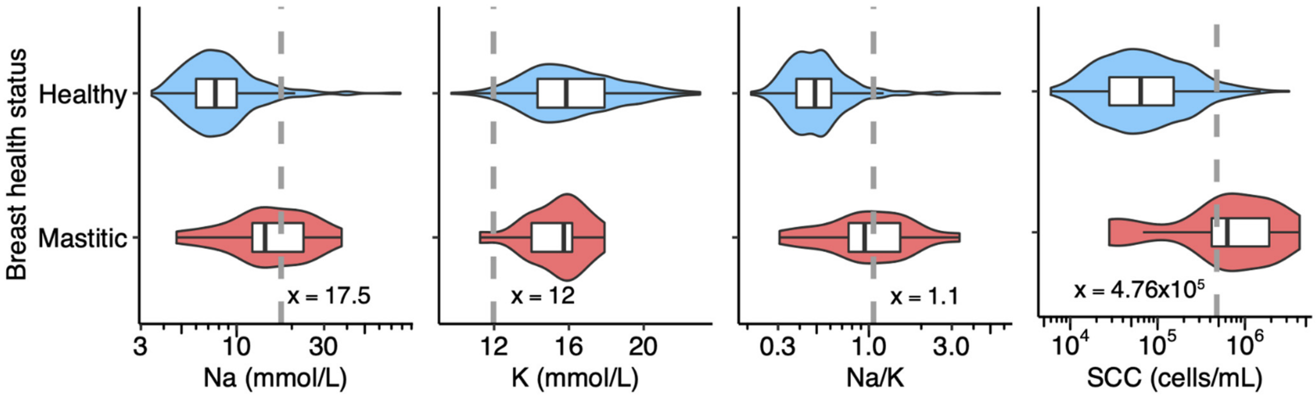

Figure S2. Analysis of biomarkers in milk produced by women with and without clinical mastitis. Distribution of biomarker concentrations in healthy (Na, K, and Na/K, *n*=468; SCC, *n*=473) and clinical mastitis (Na, K, Na/K, and SCC, *n*=16) milk samples. The inner box-and-whiskers plots show the median (middle line), first and third quartiles (lower/upper line), and 1.5 times the first and third quartiles (whiskers). The vertical dotted lines along the x-axes correspond to the logistic regression-derived biomarker thresholds for clinical mastitis.

Table S1. Self-reported perinatal- and lactation-related characteristics of participants who were healthy for the entire study and those who developed clinical mastitis.

Characteristic	Total	Healthy	Mastitis	*P*
Participants	41 (100)	31 (76)	10 (24)	-
Alcohol use during pregnancy	12 (29)	8 (26)	4 (40)	0.441
Tobacco use during pregnancy	2 (5)	2 (8)	0	>0.999
Marijuana use during pregnancy	1 (2)	1 (3)	0	>0.999
Complications during pregnancy/delivery	24 (59)	16 (52)	8 (80)	0.152
Infections during pregnancy/delivery	13 (32)	10 (32)	3 (30)	>0.999
Bacterial vaginosis	1 (2)	0	1 (10)	-
Chorioamnionitis	1 (2)	0	1 (10)	-
Group B *Streptococcus*	4 (10)	3 (10)	1 (10)	-
Herpes	2 (5)	2 (6)	0	-
Lower urinary tract infection	3 (7)	3 (10)	0	-
Other	2 (5)	2 (6)	0	-
Perceived stress during birth, median (IQR), 1-10 scale[1]	6 (3-8)	6 (3-8)	6 (5-7)	0.748
Skin-to-skin within 1 hr of birth	34 (83)	25 (81)	9 (90)	0.660
Infant feeding plans at delivery				0.376
Exclusively at the breast	30 (73)	24 (77)	6 (60)	
Mix of at the breast and expressed milk	10 (24)	6 (19)	4 (40)	
Mix of at the breast, expressed milk, and formula	1 (2)	1 (3)	0 (0)	
Infant first breastfed after delivery				0.712
<1 hr	31 (76)	23 (74)	8 (80)	
1-3 hr	8 (20)	6 (19)	2 (20)	
>3 hr	2 (5)	2 (6)	0	
Reported barriers to breastfeeding[2]	6 (15)	5 (16)	1 (10)	>0.999
Pumping milk, first 120 hours postpartum	23 (56)	16 (52)	7 (70)	0.467
Time between delivery and first pumping, median (IQR), h	72 (48-96)	84 (66-96)	72 (48-72)	0.150
Within 3 days of delivery				
Had access to a lactation consultant	19 (46)	15 (48)	4 (40)	0.727
Experienced nipple damage	20 (49)	15 (48)	5 (50)	>0.999
Infant feeding status, first week[3]				0.304
Exclusively at the breast	25 (63)	21 (68)	4 (44)	
Mix of at the breast and expressed milk	10 (25)	6 (19)	4 (44)	
Mix of at the breast and/or expressed milk with formula	5 (12)	4 (13)	1 (11)	
Exclusively fed infant human milk during entire study	35 (85)	26 (84)	9 (90)	>0.999
Exclusively fed infant at the breast during entire study	9 (22)	6 (19)	3 (30)	0.662
Nipple shield use	10 (24)	7 (23)	3 (30)	0.683
Nipple cream use	33 (80)	24 (77)	9 (90)	0.653
Infant diagnosed with tongue tie	2 (5)	1 (3)	1 (10)	0.433

IQR, interquartile range. Values are given as numeric counts and percentages or IQR in parentheses. Percentages may not sum to 100 due to rounding. *P*-values were calculated from Wilcoxon rank sum tests for continuous data and Fisher's exact tests for categorical data. [1]Stress during birth was ranked from 1 (no stress) to 10 (severe stress); [2]Reported barriers to breastfeeding included cesarean section, difficulty latching, neonatal intensive care unit stay, and infant respiratory problems; [3]Missing data from 1 participant with clinical mastitis.

nutrients

MDPI

Table S2. Na concentrations, K concentrations, Na/K, and SCC summary measurements during the first 6 weeks postpartum in lactating women without clinical mastitis (n=31).

	Week 1	Week 2	Week 3	Week 4	Week 5	Week 6
**Na (mmol/L)**						
**Left breast**	11.8 (10.3-13.5)[a]	9.5 (8.3-10.9)[b]	8.5 (7.4-9.8)[bc]	7.4 (6.5-8.5)[cd]	7.3 (6.3-8.3)[cd]	6.2 (5.4-7.1)[d]
**Right breast**	12.0 (10.5-13.8)[a]	9.5 (8.3-10.8)[b]	8.4 (7.3-9.6)[bc]	7.7 (6.8-8.9)[bcd]	6.9 (6.0-7.9)[cd]	6.5 (5.7-7.4)[d]
**Left/right mean**	11.9 (10.5-13.4)[a]	9.5 (8.4-10.7)[b]	8.5 (7.5-9.5)[bc]	7.6 (6.7-8.6)[cd]	7.1 (6.3-8.0)[de]	6.3 (5.6-7.2)[e]
**K (mmol/L)**						
**Left breast**	18.8 (18.0-19.6)[a]	16.4 (15.7-17.1)[b]	15.5 (14.9-16.2)[bc]	15.4 (14.8-16.0)[bc]	15.2 (14.6-15.8)[c]	14.8 (14.2-15.5)[c]
**Right breast**	18.6 (17.8-19.4)[a]	16.2 (15.5-16.8)[b]	15.3 (14.7-16.0)[bc]	15.6 (14.9-16.2)[bc]	15.3 (14.6-15.9)[bc]	14.6 (14.0-15.3)[c]
**Left/right mean**	18.7 (18.0-19.4)[a]	16.3 (15.7-16.9)[b]	15.4 (14.9-16.0)[c]	15.5 (14.9-16.0)[c]	15.2 (14.7-15.8)[cd]	14.7 (14.2-15.3)[d]
**Na/K**						
**Left breast**	0.63 (0.55-0.72)[a]	0.58 (0.51-0.67)[ab]	0.55 (0.48-0.63)[ab]	0.48 (0.42-0.55)[bc]	0.48 (0.42-0.55)[bc]	0.42 (0.36-0.48)[c]
**Right breast**	0.65 (0.56-0.74)[a]	0.59 (0.51-0.67)[ab]	0.55 (0.48-0.63)[abc]	0.50 (0.43-0.57)[bc]	0.45 (0.39-0.52)[c]	0.44 (0.38-0.51)[c]
**Left/right mean**	0.64 (0.57-0.72)[a]	0.58 (0.52-0.66)[ab]	0.55 (0.49-0.62)[bc]	0.49 (0.44-0.55)[cd]	0.46 (0.41-0.52)[d]	0.43 (0.38-0.48)[d]
**SCC (cells/mL, x10^5)**						
**Left breast**	0.95 (0.65-1.38)[a]	0.95 (0.65-1.39)[a]	0.85 (0.58-1.23)[a]	0.75 (0.52-1.10)[ab]	0.70 (0.48-1.03)[ab]	0.42 (0.29-0.62)[b]
**Right breast**	0.80 (0.55-1.17)[a]	0.79 (0.54-1.15)[a]	0.81 (0.55-1.17)[a]	0.88 (0.60-1.28)[a]	0.61 (0.42-0.89)[a]	0.56 (0.38-0.82)[a]
**Left/right mean**	0.87 (0.63-1.19)[a]	0.87 (0.63-1.19)[a]	0.83 (0.60-1.13)[a]	0.81 (0.59-1.12)[a]	0.66 (0.48-0.90)[ab]	0.49 (0.35-0.67)[b]

Values represent model-based estimates, means (95% confidence intervals). There were no differences in any measure between breasts (P≥0.05). Letters denote significance groups within breast over time, Tukey's method P<0.05.

Table S3. Na concentrations, K concentrations, Na/K, and SCC summary measurements of milk samples produced during clinical mastitis in the first 6 weeks of lactation.

	Week 1	Week 2	Week 3	Week 4	Week 5	Week 6
**Na (mmol/L)**						
Left breast	17.4 ± 12.9	23.3 ± 0.3	15.4 ± 3.4	7.1	-	-
Right breast	24.8	24.8 ± 17.8	16.8 ± 5.2	13.9 ± 2.5	-	8.9 ± 5.9
Left/right mean	21.1 ± 5.2	24.0 ± 1.1	16.1 ± 0.9	10.5 ± 4.9	-	-
**K (mmol/L)**						
Left breast	16.8 ± 0.9	14.7 ± 1.3	15.9 ± 0	16.4	-	-
Right breast	16.1	13.3 ± 2.9	13.9 ± 0.2	17.4 ± 0.7	-	14.6 ± 1.1
Left/right mean	16.4 ± 0.5	14.0 ± 1.0	14.9 ± 1.4	16.9 ± 0.7	-	-
**Na/K**						
Left breast	1.06 ± 0.83	1.59 ± 0.12	0.97 ± 0.21	0.43	-	-
Right breast	1.54	2.06 ± 1.79	1.19 ± 0.36	0.80 ± 0.18	-	0.63 ± 0.45
Left/right mean	1.30 ± 0.34	1.82 ± 0.33	1.09 ± 0.16	0.62 ± 0.26	-	-
**SCC (cells/mL, x10^5)**						
Left breast	10.20 ± 14.03	13.83 ± 12.47	14.54 ± 9.12	0.68	-	-
Right breast	4.99	3.62 ± 4.70	9.25 ± 5.01	22.37 ± 28.21	-	11.80 ± 9.63
Left/right mean	7.60 ± 3.68	8.73 ± 7.22	11.90 ± 3.74	1.23 ± 0.78	-	-

Values represent means ± standard deviations (when multiple observations occur). Milk samples were collected from 10 participants. In total, there were 16 milk samples: 3 in week 1, 4 in week 2, 4 in week 3, 3 in week 4, 0 in week 5, 2 in week 6.
